# Nitric Oxide Production in Professional Skiers During Physical Activity at Maximum Load

**DOI:** 10.3389/fcvm.2020.582021

**Published:** 2020-12-14

**Authors:** Olga I. Parshukova, Nina G. Varlamova, Evgeny R. Bojko

**Affiliations:** Institute of Physiology at Komi Science Center of the Ural Branch of the Russian Academy of Sciences, Syktyvkar, Russia

**Keywords:** nitric oxide, cardiovascular system, endothelial dysfunction, exercise test on a cycle ergometer, cross-country skiers

## Abstract

The purpose of this study is to assess the production of nitric oxide in professional cross-country skiers with normotensive and hypertensive responses to physical activity at maximum load. The observation group included professional cross-country skiers (22.2 ± 7.1 years, = 107) who were current members of the national team of the Komi Republic. All the examined athletes performed the exercise test on a cycle ergometer “until exhaustion.” The following parameters were determined for each participant while they were sitting at rest, while at their anaerobic threshold level, during peak load, and during the recovery period (5th min): systolic blood pressure, diastolic blood pressure, heart rate, and the level of stable nitric oxide metabolites (nitrites, nitrates) in capillary blood samples. According to the blood pressure results, the cross-country skiers were divided into two groups. Group I included athletes with a normotensive response to stress. Group II was composed of individuals with a hypertensive response to stress. During the performance of the test “until exhaustion,” a significant (*p* < 0.05) increase in the amount of stable metabolites of nitric oxide was observed in the group of athletes with a normotensive response to the load compared with the group with a hypertensive response to the load. In athletes with a normotensive reaction to the load during exercise at maximum load and in the early recovery period, nitrate was prioritized in the regulation of vascular tone. The exercise test on a cycle ergometer “until exhaustion,” combined with the assessment of the levels of stable nitric oxide metabolites in plasma, can be considered a test for the early diagnosis of endothelial dysfunction in professional athletes.

## Introduction

One of the factors limiting the functional capabilities of the bodies of athletes is overstraining of the cardiovascular system (1). Physical exercise leads to a progressive increase in heart rate (HR), which subsequently increases blood flow and vascular shear stress. Nitric oxide (NO), a signaling molecule, plays an important role in the regulation of many physiological functions, including regulating circulation and blood pressure by vasodilation (2, 3). The potential mechanisms through which vascular control may be beneficially modified in response to repeated exposure to shear stress include increased endothelium-dependent dilator capacity (4) and higher production of endothelial NO and endothelial NO synthase (eNOS) expression (5). In athletes, peripheral vasoconstriction during maximal exercise is prevented by improved endothelium-dependent dilator capacity (as indicated by plasma levels of nitrite oxide), which may increase skeletal muscle oxidative capacity during exercise (5). An imbalance in the NO synthesis system and formation of endothelial dysfunction play an important role in the development of cardiovascular pathology. Prolonged and intense aerobic training can result in a decrease in NO bioavailability, which weakens endothelial-dependent vasodilation (6). According to the literature (7), systolic blood pressure (SBP) >200 mmHg and/or diastolic pressure (DBP) >100 mmHg serve as a predictor for the termination of the exercise test “until exhaustion” in professional cross-country skiers.

Impaired NO generation is important in the development of endothelial dysfunction and hypertension (3, 6). Endothelial dysfunction leads to prioritized production of vasoconstrictor substances (tissue AII, endothelin, and thromboxane A2), decreased production of depressant compounds (bradykinin, NO, and prostacyclin), and an inadequate regulatory response of the vascular wall. As a result, endothelium-dependent vasoconstriction prevails over endothelium-dependent vasodilation (8).

Thus, the mechanisms of the development of endothelial dysfunction may vary, and the significance of the contribution of the features of NO synthesis as an early marker remains unresolved. According to the literature (9), SBP increases significantly and proportionally to workload increases during exercise tests in healthy adults, which can be useful in the diagnosis of cardiovascular diseases. In this regard, it can be assumed that the cardiovascular response of the body of a professional athlete to physical load of maximum load may depend not only on the mechanisms of hypertension formation but also on the specific features of NO synthesis.

The purpose of this study was to assess NO production in professional cross-country skiers with normotensive and hypertensive responses to physical activity at maximum load.

## Methods

### Ethical Approval

The experimental design and protocol of the study were approved by the Ethics Committee of the Institute of Physiology, Russian Academy of Sciences, Syktyvkar. The study conformed to the Code of Ethics of the World Medical Association (Declaration of Helsinki). The participants were given all the information about the experimental protocol, experimental procedures, and possible risks and discomforts associated with performing the exercise test on a cycle ergometer “until exhaustion.” The participants were informed that they were free to withdraw from the study at any time and without consequence. After the required explanations, the volunteers gave their written informed consent for participating in the experiment.

### Participants

The observation group included well-trained cross-country skiers (*n* = 107 males, 22.2 ± 7.1 years old) who were current members of the national team of the Komi Republic, had no signs or history of chronic diseases, and volunteered to participate in this study. All athletes had extensive experience in endurance events and had a minimum of 5 years of cross-country skiing practice as part of their main training schedule.

### Experimental Protocol

The study was performed during the morning after a breakfast low in nitrates; therefore, all of the volunteers consumed a standardized meal (400–420 kcal) consisting of (in units of the percentage of the total energy supplied by the entire meal, En%) 78 En% carbohydrate, 14 En% fat, and 8 En% protein the night before the assessment. The participants' dietary intake was assessed using a food frequency questionnaire. Calories consumed at breakfast were not normalized by body weight. The low-nitrate dinner excluded foods and drinks that are the main sources of nitrates in human food (meat and fish products, vegetables (mainly beets, leafy green vegetables), marinades, spirits, fruit, and mineral drinks).

**Figure 1:**
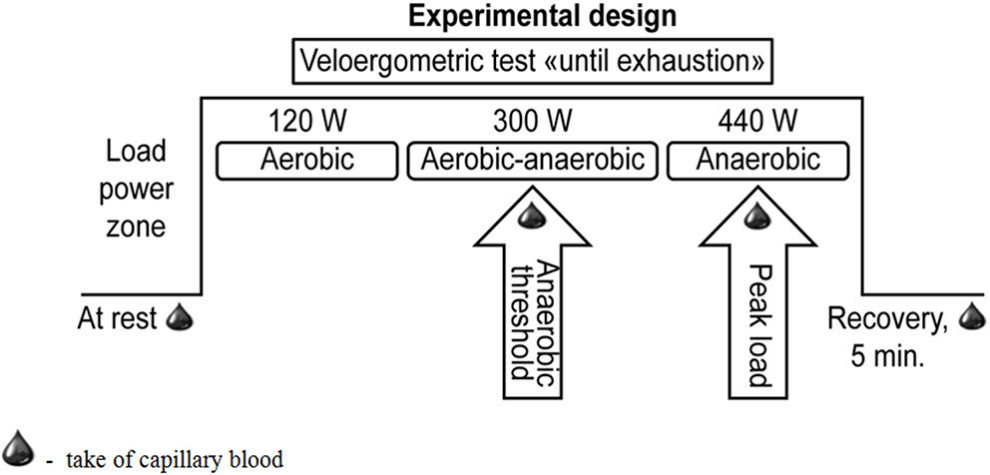
Overview of the study design incorporating the exercise test on a cycle ergometer “until exhaustion”.

In each of the examined athletes, the following parameters were determined when sitting at rest, at the anaerobic threshold level, during peak load, and during the recovery period (5th min) (Figure 1): SBP, DBP, heart rate (HR), and the level of stable metabolites of NO in capillary blood samples. Blood pressure was measured by the Korotkov method on the right arm. Heart rate data were obtained from test protocols. The pulse pressure (PP) value was calculated as the difference between SBP and DBP. The maximum oxygen consumption per kilogram of body weight (VO_2_ max/kg) was estimated.

According to the blood pressure results, the cross-country skiers were divided into two groups. Group I included athletes with a normotensive response to the load (SBP at a peak load of up to 200 mmHg and/or DBP at a peak load of up to 100 mmHg). Group II was composed of individuals with a hypertensive response to the load (SBP at a peak load of >200 mmHg and/or DBP at a peak load of >100 mmHg). The characteristics of the examined groups of athletes are presented in Table 1.

**Table 1:** Characteristics of the subjects by group, Me ± SD.

**Parameters**	**Group I (*n* = 50)**	**Group II (*n* = 57)**
Age, years	21.4 ± 6.0	22.7 ± 7.6
Weight, kg	69.5 ± 3.6	70.9 ± 3.7
Height, cm	176.2 ± 3.5	179.8 ± 4.4
Load power, W	363.2 ± 38.8^**^	339.4 ± 38.3^**^
Maximal oxygen uptake/kg, ml/min/kg	64.4 ± 5.6^*^	61.0 ± 7.8^*^

*Statistical significance levels between groups*:

* *p < 0.05*;

** *p < 0.01*.

### Exercise Test on a Cycle Ergometer “Until Exhaustion”

Aerobic capacity (VO_2_ max) testing was performed on an ergometer bike (“Ergoselect-100,” Ergoline GmbH, Germany) in the “breath by breath” mode, and the indicators were averaged over 15-s segments. The protocol included 1 min of cycling without load (for adaptation) followed by stepwise load increases of 40 W in 2-min increments starting with an initial load of 120 W. The pedaling speed was 60 rpm. The anaerobic threshold (AT) was determined by reaching a respiratory coefficient of one (10, 11).

### Determination of NO_x_

The NO levels in the plasma were measured indirectly by evaluating the stable metabolites of NO, including nitrites [NO(_2_)(–)] and nitrates [NO(_3_)(–)], which were presorted as an index (NO_x_) of NO production using the Griess reaction. NO is rapidly converted to nitrite and nitrate, the terminal products of NO in human plasma. A strong correlation between endogenous NO production and NO_x_ levels in plasma has been reported (12). Briefly, each of the examined athletes was collected capillary blood samples when sitting at rest, at the anaerobic threshold level, during peak load, and during the recovery period (5th min). One-milliliter blood samples were collected into tubes with heparin and immediately centrifuged for 20 min at 2,500 g. The separated plasma was aliquoted and stored at −40°C until analysis. Samples were measured twice against a standard nitrite curve with a known concentration. Plasma samples were deproteinized by precipitation in ethanol. After centrifugation, the supernatants were incubated for 30 min at 37°C with vanadium chloride to convert nitrate to nitrite. Next, the samples were mixed with the Griess reagent, and the light absorbance was measured at a wavelength of 540 nm using a Spectronic Genesys-6 Spectrophotometer (Thermo Electron Scientific Instruments LLC, Madison, WI USA). Total nitrite was then measured using the Griess reagent. The plasma nitrate concentration was calculated by subtracting the primary nitrite concentration from the total nitrite concentration. All chemicals used for NO determination were obtained from Sigma (St. Louis, MO, USA). The detection limit for NO was 0.001 μmol/l. The NO_3_/NO_2_ index was calculated as the ratio between NO_3_ and NO_2_.

### Statistical Analysis

Statistical analyses were performed using Statistica software (version 6.0, StatSoft Inc., 2001, USA). Descriptive statistics were used to calculate the mean and standard deviation (SD). Differences in the dynamics of each parameter were tested by Friedman's ANOVA. The significance of variation between the groups was estimated by the Wilcoxon-test. The correlation coefficients between two variables were determined by Spearman rank analysis. A value of *p* < 0.05 was accepted as statistically significant.

## Results

### Hemodynamic Parameters

SBP at rest was increased (*p* < 0.001) in individuals with a hypertensive response to the load compared with the group with a normotensive response to the load. When performing the test “until exhaustion,” all cross-country skiers showed a statistically significant increase in SBP during the passage of the anaerobic threshold (*p* = 0.001) compared with indicators at rest. During the maximum load (peak load), cross-country skiers continued to increase their SBP compared with the period of passage of the AT. At the level of the AT and peak load, the group that had a hypertensive response to the load was characterized by higher values of SBP (*p* < 0.001). During the recovery period, SBP-values significantly decreased in both groups (*p* < 0.001) (Table 2).

**Table 2:** Hemodynamic parameters at different stages of the load in professional cross-country skiers, Me ± SD.

**Parameters**		**Stages of the load**			
		**At rest**	**Anaerobic threshold**	**Peak load**	**Recovery**
Systolic blood pressure, mmHg	I	113.8 ± 6.9^***^	175.6 ± 11.5^***^^###^	1181.5 ± 13.6^***^^#^	124.1 ± 14.1^###^
	II	123.4 ± 11.8	192.9 ± 17.7^###^	199.3 ± 11.5	128.8 ± 12.2^###^
Diastolic blood pressure, mmHg	I	77.6 ± 8,3	75.7 ± 15,5	78.1 ± 14,1	64.7 ± 10,9^**^^###^
	II	80.5 ± 9.5	82.0 ± 19.0	80.3 ± 26.4	72.2 ± 13.2^##^
Pulse pressure, mmHg	I	36.2 ± 9.1^**^	99.9 ± 15.5^*^^###^	103.4 ± 18.9^*^	59.4 ± 15.1^###^
	II	42.9 ± 11.4	110.9 ± 27.6^###^	119.0 ± 30.6	56.6 ± 17.3^###^
Heart rate, beats/min	I	65.6 ± 10.5	171.5 ± 13.9^###^	189.9 ± 8.8^*^^###^	105.7 ± 11.5^**^^###^
	II	69.4 ± 14.9	169.8 ± 13.4^###^	185.3 ± 8.8^###^	98.8 ± 13.0^###^

*Statistical significance levels between groups in each stages of the load*:

* *p < 0.05*;

** *p < 0.01*;

*** *p < 0.001*.

*Statistical significance levels with the previous stage of the load*:

# *p < 0.05*;

## *p < 0.01*;

### *p < 0.001*.

DBP-values at rest, AT, and peak load did not significantly change in the study groups. However, during the recovery period, DBP was significantly (*p* < 0.001–0.01) decreased in both groups. Moreover, the decrease in DBP in the group with a normotensive response to the load was more pronounced than that in the opposite group (*p* < 0.01).

The dynamics of changes in the PP-value in the two groups were similar; specifically, significant increases during the period of the AT passage and a decrease 5 min after the end of the test were noted (*p* < 0.001). However, in the group with a hypertensive reaction to the load at rest, AT, and peak load, the value of this indicator was increased compared to a group of people with a normotensive reaction to the load (*p* < 0.01–0.05).

A significant (*p* < 0.001) increase in heart rate was found in both groups of cross-country skiers during the passage of the AT of the load compared with indicators at rest. A continued increase in heart rate was noted at the peak of the load compared with the period of passage of the AT (*p* < 0.001). During this load period, group I was characterized by increased heart rates compared to group II (*p* < 0.05). During recovery, athletes in the study groups showed a statistically significant decrease in heart rate compared with that noted during the peak load (*p* < 0.001).

### Nitric Oxide Levels

The values of NO_x_ at rest did not exhibit statistically significant differences between the study groups. In subjects with a normotensive response to the load, an increase in NO_x_ was detected during the passage of the AT compared with values at rest (*p* < 0.05). During periods of peak load and recovery in this group, NO_x_ tended to decrease (Table 3). In the group with a hypertensive reaction to the load during the passage of the AT, the level of NO_x_ in the blood tended to increase. At the peak load and recovery, the group with a normotensive response to the load was characterized by higher values of NO_x_ (*p* < 0.01).

**Table 3:** Nitric oxide levels at different stages of the load in professional cross-country skiers, Me ± SD (μmol/L).

**Parameters**		**Stages of the load**			
		**At rest**	**Anaerobic threshold**	**Peak load**	**Recovery**
NOx	I	25.7 ± 10.1	32.9 ± 13.9^#^	29.8 ± 12.1^**^	29.5 ± 9.8^**^
	II	25.3 ± 11.5	27.2 ± 11.4	24.8 ± 10.6	24.9 ± 9.3
NO2	I	12.2 ± 6.3	13.2 ± 7.7^#^	13.7 ± 5.0	11.9 ± 5.7^#^^**^
	II	11.7 ± 6.1	12.6 ± 6.6	13.5 ± 7.1	13.8 ± 6.6
NO3	I	13.4 ± 7.9	19.7 ± 11.8^#^	16.1 ± 11.9^*^	17.6 ± 10.3^***^
	II	13.5 ± 8.4	14.7 ± 8.1	11.2 ± 7.4^#^	11.2 ± 6.8

*Statistical significance levels between groups in each stages of the load*:

* *p < 0.05*;

** *p < 0.01*;

*** *p < 0.001*.

*Statistical significance levels with the previous stage of the load*:

# *p < 0.05*.

The NO_2_ value between the study groups at rest, AT, and peak load did not exhibit statistically significant changes. An increase in NO_2_ was detected in group I during periods of the AT passage compared with values at rest (*p* < 0.05). It should be noted that during recovery 5 min after the end of the test, different dynamics of changes in the level of NO_2_ were observed. An increase in NO_2_ was detected in group II, while a decrease was noted in group I (*p* < 0.01).

**Figure 2:**
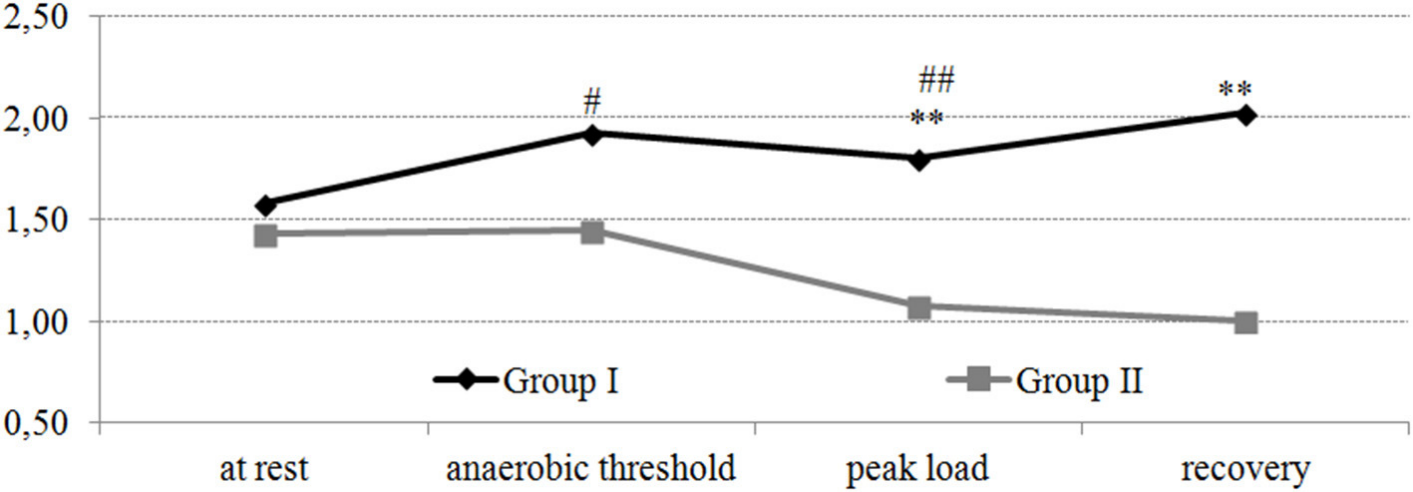
Dynamics of the NO_3_/NO_2_ index in professional cross-country skiers during the exercise test on a cycle ergometer “until exhaustion.” Statistical significance levels between groups: *p < 0.05; **p < 0.01; ***p < 0.001. Statistical significance levels between stages of the load: ^#^*p* < 0.05; ^##^*p* < 0.01; ^###^*p* < 0.001.

A statistically significant (*p* < 0.05) increase in NO_3_ was noted in group I during the passage of the AT compared with the values at rest. In the group with a hypertensive reaction to the load at the peak of the load, the value of NO_3_ was lower compared with the values at the AT (*p* < 0.05). At the same time, at both the peak of the load and recovery timepoints, the value of NO_3_ was lower in group II compared with group I (*p* < 0.05–0.001). It should be noted that for the subjects with a normotensive response to the load, an increase in the NO_3_/NO_2_ index was observed during the test “until exhaustion” (Figure 2). In the athletes with a hypertensive reaction to the load, a decrease in the NO_3_/NO_2_ index was observed during the peak load and during the recovery period.

Thus, in athletes with different hemodynamic responses to the load, the opposite dynamics of changes in nitric oxide indicators were noted during the test “until exhaustion.”

### Interrelationship of Hemodynamic Parameters and Nitric Oxide Levels

After conducting a correlation analysis in the group with normotensive reactions, a negative relationship between NO_3_ values and SBP during recovery was revealed (*r* = −0.38, *p* < 0.05). At the peak load, positive relationships were observed between DBP and NO_x_, NO_3_, and the NO_3_/NO_2_ index (*r* = 0.48, *p* < 0.01; *r* = 0.59, *p* < 0.001; *r* = 0.53, *p* < 0.001, respectively). In the group with a normotensive response to the load, negative relationships among NO_3_, NO_3_/NO_2_, and pulse pressure indicators at different stages of the test were revealed. It should be noted that the relationship between heart rate values and indicators of nitric oxide at rest was negative (Table 4).

**Table 4:** Correlations between biochemical and hemodynamic parameters at different stages of the load in professional cross-country skiers in groups I and II.

**Stages of the load**	**Parameters**	**Spearman rank order correlations**			
		**NOx**	**NO2**	**NO3**	**NO3/NO2**
Before load, at rest	SBP				0.29^*^
	DBP				
	PP	−0.30^*^		–0.41^**^	
	HR	–0.42^**^		–0.43^**^	−0.29^*^
Anaerobic threshold	SBP				
	DBP				
	PP		−0.29^*^		
	HR			–0.41^**^	
Peak load	SBP				
	DBP	0.48^**^	–0.33^*^	0.59^***^	0.53^***^
	PP		0.35^*^	–0.52^***^	–0.53^***^
	HR				
Recovery	SBP			–0.38^*^	
	DBP				
	PP		0.28^*^		–0.38^*^
	HR				

*Statistical significance levels*:

* *p < 0.05*;

** *p < 0.01*;

*** *p < 0.001*.

*SBP, systolic blood pressure; DBP, diastolic blood pressure; PP, pulse pressure; HR, heart rate*.

*Bold font indicates data of group athletes with a normotensive response to the load*.

*Italic font indicates data of group athletes with a hypertensive response to the load*.

In the group with a hypertensive reaction to the load, a negative relationship was noted between NO_2_ and DBP-values during the peak load (*r* = −0.33, *p* < 0.05), and a positive correlation was noted between the NO_3_/NO_2_ index and SBP at rest. Unlike in group I, a correlation between the PP and the level of NO_2_ was noted in the group with a hypertensive response to the load; the correlation was negative (*r* = −0.29, *p* < 0.05) during the anaerobic threshold and positive at peak load and during recovery (*r* = 0.35, *p* < 0.05; *r* = 0.28, *p* < 0.05, respectively). In this group, the heart rate indicator showed a correlation only with the NO_3_/NO_2_ index at rest (*r* = −0.29, *p* < 0.05).

Thus, athletes with a normotensive response to the load revealed more statistically significant and closer relationships between hemodynamic parameters and the level of nitric oxide in the blood. Typically, three indicators (NO, NO_3_, NO_3_/NO_2_) participate in the regulation of vascular tone at the peak of the load; however, only NO_2_ is involved in individuals with a hypertensive reaction to the load. The priority biochemical indicator is NO_3_ for athletes from group I and NO_2_ for athletes from group II.

## Discussion

In the athletes examined in this study, the SBP at rest corresponded to the norm, and the DBP at rest was higher than that reported in literature based data obtained from students from a physical education department (64.0 ± 4.7 mmHg) (13). An increase in DBP at rest in both of the groups examined in this study could be associated with prolonged training in open, cold air, which could lead to an increase in peripheral vascular resistance (14). According to the literature (15), even short-term (1 h) cold exposure induces toward hypercoagulation in young healthy people, which can also cause a higher level of DBP. Additionally, cold air can indirectly lead to an increase in cardiovascular risks through its effect on the sympathetic and renin-angiotensin systems, blood pressure, and risk factors for atherosclerosis, such as blood viscosity and the amount of fibrinogen, lipids, and uric acid (16). In general, people who have been in open, cold air for a long time are characterized by increased blood pressure (17), which probably affects the value of SBP during exercise and limits its tolerance.

It is known that blood pressure increases during a load test, and the degree of its growth is proportional to the intensity and severity of the work performed (9). However, according to our data, in athletes with a normotensive response to the load, the power of the performed load was increased compared with that noted in a group of athletes with a hypertensive response to the load. Additionally, in athletes from group II, PP was higher at rest, the AT and peak load. These results also indicate the effect of the hypertensive component.

It is known (18) that in the body of professional athletes under intense and strenuous physical exertion, oxidative stress occurs, leading to the accumulation of lipid peroxidation products, including free radicals. Oxidative stress is the main reason for the decrease in the activity of NO synthase (NOS) through a decrease in the availability of the co-factor NOS-tetrahydrobiopterin and, subsequently, the inhibition of the enzymatic synthesis of NO. With a decrease in the level of NO in the tissues, the adaptive capacity of the body decreases, and pathological changes in metabolism are observed, leading to diseases. There is an opinion that the primary cause of the pathogenesis of coronary heart disease and atherosclerotic vascular damage is a deficiency of NO in the vascular endothelium and myocardium (19). In our study, a decrease in NO_x_ levels was observed during exercise at maximum load, in a group of athletes with a hypertensive response to the load, which may suggest a decrease in the enzymatic synthesis of NO. This finding also confirms the previously obtained information that, in the pathology of the cardiovascular system, a decrease in NO levels in tissues is observed (20).

In particular, in most clinical studies (21–23), in patients with stage II hypertension (HT), a decrease in NO_x_ levels was detected, which is quite common in the context of endothelial dysfunction. Deficiency of endothelial NO can be caused by several factors: a decrease in eNOS activity (21), destruction or capture of NO by free radicals, and/or a weakening of the effect of NO on smooth muscle (22). Reduced vasodilator function of the endothelium is an important link in the pathogenesis of HT. However, in a number of studies on hypertension, an increase in NO metabolites was found, which appeared during tress, humoral system activation, or a more severe course of hypertension (23).

According to our data, the athletes with a hypertensive reaction to the load show a decrease in the level of NO_3_ in the blood, and NO_2_ tended to increase during the peak and recovery periods. In these periods of the study, we also revealed correlations among NO_2_, DBP, and PP. That is, the higher the NO_2_, the lower the DBP-value and the higher the PP-value. At the same time, there were no correlations between NO_3_ and hemodynamic parameters.

Until recently, NO_2_ and NO_3_ were considered relatively inert intermediate products of NO oxidation, and these molecules are synthesized in the body by the NO synthase enzyme. However, in recent years, unique properties of NO_2_ have been discovered, making it possible to recognize it as the most important biologically active signaling molecule (24). The significant vasodilator response observed in *in vivo* and *in vitro* experiments on the introduction of NO_2_ solutions suggested that it could be an alternative source of NO (25). Information had also been reported on the involvement of NO_2_ in the adaptation to physiological hypoxia conditions, such as those caused by physical exertion (26). Modern ideas about nitrite-dependent mechanisms of adaptation to hypoxia are based on data on the involvement of NO_2_ in oxygen-dependent and hypoxic-dependent nitrite reductase processes. The NO released as a result of these processes is involved in the regulation of vascular tone, modulation of mitochondrial redox reactions (27), changes in the sensitivity of cardiac contractile proteins to oxygen (28) and calcium ions (29), and inhibition of inducible NOS. Due to the cyclic metabolic transformations of NO, NO_2_, and NO_3_, the optimal level of NO necessary for the normal functioning of the cardiovascular system under conditions of impaired functioning of NO synthases is maintained. On the other hand, excess NO is removed via the formation of an NO depot in the form of NO_2_ that protects tissues from oxidative and nitrosative stress. It has now been established that the source of vasoactive NO is also NO_2_, which is always present in the blood and can be reduced to NO enzymatically, under the influence of xanthine oxidoreductase, and non-enzymatically, under conditions of low pH and pO_2_ (30). Nitrate is sequentially reduced to nitrite and nitric oxide, which activate soluble guanylate cyclase (31). In particular, exercise has been shown to increase plasma nitrite levels by increasing NO synthesis in endothelial cells (32, 33). Plasma nitrite is gradually oxidized to nitrate, a process that is greatly accelerated by the presence of heme proteins (34). Nitrate is stable in plasma until it is excreted in the urine. Circulating nitrite rather than nitrate reflects endothelial-dependent NO synthesis (35). The increased production of NO during exercise is probably controlled by increasing nitrate excretion as a possible mechanism to control plasmatic homeostasis (36). When oxygen tension decreases, the reduction of nitrite by deoxyhemoglobin produces NO. Erythrocyte NO generation and output, along with the oxygen concentration gradient, could be related to the role of nitrite bound to erythrocytes in vasodilatation processes in response to hypoxia. Thus, it can be assumed that in athletes with a hypertensive response to exercise, NO_2_ was used as an NO precursor for vasodilation, but it is possible that this compensatory mechanism for regulating cardiovascular tone was insufficient.

Thus, in the group of athletes with a hypertensive reaction to the load, an imbalance was established in the NO synthesis system, which led to a significant increase in blood pressure during exercise at maximum load. The observed imbalance in the NO synthesis system during exercise can subsequently lead to the occurrence of endothelial dysfunction and play an important role in the development of hypertension which will also be registered at rest, and cardiovascular pathology.

## Conclusions

At rest, in athletes with normotensive and hypertensive responses to the load, the hemodynamic parameters and the level of stable metabolites of nitric oxide and their amounts in the blood corresponded to generally accepted standards, which was not informative for predicting endothelial dysfunction. However, during the performance of the test “until exhaustion” by athletes in the group with a normotensive response to the load, a significant (*p* < 0.05) increase in the amount of stable metabolites of nitric oxide was observed compared with the group with a hypertensive response to the load. In cross-country skiers with a normotensive reaction to the load during exercise at maximum load and in the early recovery period, nitrate is primarily involved in the regulation of vascular tone. The development of hypertensive reactions in these athletes during the test was due to nitrate deficiency. Thus, the exercise test on a cycle ergometer “until exhaustion” combined with the assessment of the levels of stable nitric oxide metabolites in plasma can be considered a test for the early diagnosis of endothelial dysfunction in professional athletes.

## Data Availability Statement

The raw data supporting the conclusions of this article will be made available by the authors, without undue reservation.

## Ethics Statement

The studies involving human participants were reviewed and approved by Ethics Committee of the Institute of Physiology, Russian Academy of Sciences, Syktyvkar. The patients/participants provided their written informed consent to participate in this study.

## Author Contributions

OP participated in the experimental procedure, carried out the biochemical studies, performed the statistical analysis, and drafted the manuscript. NV participated in the experimental procedure, involved in data collection, and drafted the manuscript. BE oversaw the experimental procedures, provided coordination, and helped with drafting of the manuscript. All authors have read and approved the final version of the manuscript, agreed with the order of presentation of the authors, and participated in designing the experiment.

## Conflict of Interest

The authors declare that the research was conducted in the absence of any commercial or financial relationships that could be construed as a potential conflict of interest.
